# Pre-segmented 2-Step IMRT with subsequent direct machine parameter optimisation – a planning study

**DOI:** 10.1186/1748-717X-3-38

**Published:** 2008-11-06

**Authors:** Klaus Bratengeier, Jürgen Meyer, Michael Flentje

**Affiliations:** 1 Klinik und Poliklinik für Strahlentherapie, Universität Würzburg, Josef-Schneider-Str. 11, 97080 Würzburg, Germany; 2 Department of Physics and Astronomy, University of Canterbury, Private Bag 4800, Christchurch 8140, New Zealand

## Abstract

**Background:**

Modern intensity modulated radiotherapy (IMRT) mostly uses iterative optimisation methods. The integration of machine parameters into the optimisation process of step and shoot leaf positions has been shown to be successful. For IMRT segmentation algorithms based on the analysis of the geometrical structure of the planning target volumes (PTV) and the organs at risk (OAR), the potential of such procedures has not yet been fully explored. In this work, 2-Step IMRT was combined with subsequent direct machine parameter optimisation (DMPO-Raysearch Laboratories, Sweden) to investigate this potential.

**Methods:**

In a planning study DMPO on a commercial planning system was compared with manual primary 2-Step IMRT segment generation followed by DMPO optimisation. 15 clinical cases and the ESTRO Quasimodo phantom were employed. Both the same number of optimisation steps and the same set of objective values were used. The plans were compared with a clinical DMPO reference plan and a traditional IMRT plan based on fluence optimisation and consequent segmentation. The composite objective value (the weighted sum of quadratic deviations of the objective values and the related points in the dose volume histogram) was used as a measure for the plan quality. Additionally, a more extended set of parameters was used for the breast cases to compare the plans.

**Results:**

The plans with segments pre-defined with 2-Step IMRT were slightly superior to DMPO alone in the majority of cases. The composite objective value tended to be even lower for a smaller number of segments. The total number of monitor units was slightly higher than for the DMPO-plans. Traditional IMRT fluence optimisation with subsequent segmentation could not compete.

**Conclusion:**

2-Step IMRT segmentation is suitable as starting point for further DMPO optimisation and, in general, results in less complex plans which are equal or superior to plans generated by DMPO alone.

## Background

Historically, inverse planning algorithms [1], derived from tomographic calculations, have played a more important role than "forward planning" techniques [2-4] for IMRT. In contrast to traditional "trial and error" methods for three-dimensional conformal radiotherapy (3DCRT), progressive "forward planning" techniques for IMRT analyse the geometrical constellation to create beam segments. The weights of these segments can then be optimised in the same fashion as in conventional inverse IMRT planning. A few hospitals have specialised in such "forward planning" and can compete with "inverse planning" techniques [5-8]. Their approach is presumed not to be as conformal and flexible as an inverse planning based one; often "forward planning" is used in terms of class solutions. However, algorithms such as 2-Step IMRT [9,10] are utilized for a variety of tumour localizations [6,11]. 2-Step IMRT is a segmentation technique, which creates segments, reflecting the shape of the tumour and OARs but also highly weighted narrow segments close to critical structures to obtain steep dose gradients [12]. On the other hand, contemporary "inverse planning" uses iterative optimisation elements. The latest generation of planning systems integrates machine parameters in the iterative optimisation process, such as HYPERION [13] and direct aperture optimisation (DAO) [14-16]. The Pinnacle3^® ^planning system refers to it as "direct machine parameter optimization" ("DMPO", Raysearch™ laboratories). Such algorithms are clearly superior to those with sequencing after a complete fluence optimization process [17]. Fine-tuning of the segment parameters, such as with DMPO, could possibly also enhance "forward planning" techniques. The aim of this work is to explore whether a technique like 2-Step IMRT could not only compete with a former generation planning systems (fluence optimisation followed by subsequent segmentation) as shown before [6], but also compete with a planning system of the latest generation where segmentation is integrated in the optimisation procedure such as with DMPO. A further motivation for this planning study is to investigate the possibility of daily ad-hoc adaptation of IMRT-plans[10,18] based on 2-Step IMRT. This could only be useful if the primary plan can concur with the results of up to date IMRT planning systems. Without an initial IMRT plan of sufficient quality, all adaptive efforts would be non-optimal. With this in mind, it should be noted that the adaptation process itself is not subject of this paper.

## Methods

### Planning procedure DMPO-25, DMPO-50

The IMRT optimization in Pinnacle with DMPO starts with a conformal beam of uniform intensity followed by four steps of fluence optimization. This is followed by a step that includes machine parameters: Leaf-positions and segment weights are varied within the limits of the linear accelerator. Two such DMPO plans were generated for each case used in this paper. The first with only 25 optimisation steps (DMPO-25), the second with additional 25 steps (DMPO-50), to be able to track the speed of convergence. A further increase of the number of steps does not necessarily lead to better results[19,20].

For both DMPO-plans slightly higher segment numbers were allowed to give them a chance to generate better plans than in combination with 2-Step IMRT (see next section).

### Planning procedure 2S-DMPO-25, 2S-DMPO-50

For 2-Step IMRT, the segments were manually shaped according to the 2-Step IMRT-algorithm, which is described in detail in the preceding publications[6,9-11,19]. Several classes (orders) of beam segments were defined. All are generated by use of the beams-eye-view (BEV)-projections of PTV and OARs

**Figure 1:**
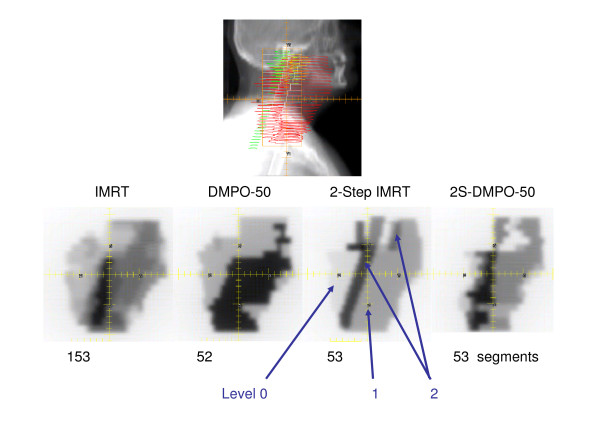
**Head and neck case – DRR and fluences**. Example (head and neck case B_2_) of digital reconstructed radiography (DRR) with projections of the most extended PTV and the spinal cord. (upper row). Fluences for several techniques for the same gantry angle at a head and neck case (lower row). Indicated are the segment numbers of all fields and the 2-Step IMRT-levels defined in Bratengeier et al. (2007)[6]

• 0^th ^order "conformal" segments include the target volume neglecting any OAR

• 1^st ^order "OAR-sparing" segments include the PTV excluding the OAR

• 2^nd ^order "narrow" segments were defined adjacent to the OAR. Their definition is more complex and needs 3D information [10]

(see i.e. the fluence levels in Figure 1). The segment weights were optimised by means of the planning system[6]. Segments with less than one monitor unit were discarded. With this step, the number of segments was drastically reduced. This situation was considered as the start condition for subsequent optimization. Subsequently, the DAO algorithm was applied ("DMPO" without "beam reset") for 25 optimisation steps (2S-DMPO-25). In a second run, further 25 steps (2S-DMPO-50) were applied (again after discarding few segments with weights of less than 1 MU). In this manner, 2-Step IMRT replaced the initial situation – the fluence optimisation – of the commercial "DMPO".

### Planning procedure "IMRT"

For comparison, the "IMRT" algorithm of the planning system – detailed fluence optimisation followed by the segmentation – was used as benchmark.

### Patient models

This planning study utilized Pinnacle3^®^(Philips/ADAC laboratories) for all planning steps. 15 clinical cases were chosen: A: 4 prostates with simultaneous integrated boost (SIB), B: 4 head and neck (3 cases with SIB), C: 4 breast with parasternal lymph nodes or with protruding thoracic wall, D: 1 angiosarcoma of the scalp, E: 1 bone-metastasis partially surrounding the spine, F: 1 bone metastasis with SIB. Furthermore the ESTRO Quasimodo-Phantom[21] (Q) was used in two ways: with 9 and 15 equidistant irradiation angles. This oval phantom (20 cm × 37 cm, length 16 cm) contains a horse-shoe-shaped PTV (max. diameter 16 cm) surrounding a circular OAR (diameter 5 cm) at a distance of 0.5 cm. The body outside the PTV (Body\PTV) is considered as organ at risk.

### Planning parameters and optimisation goals

For all clinical cases except the breast tumours 9 equidistant fields were employed starting from 180° (prostate cases) or 0° (other cases). For the left breast beams at angles 240°, 250°, 265°, 285°, 310°, 345°, 10°, 30°, 45°, 55° were applied and for the right breast 305°, 315°, 330°, 350°, 15°, 50°, 75°, 95°, 110° 120°. One of the breast cases (C_2_) was supplied by the group of Fogliata and Cozzi (case #3)[22] to be able to compare with international standards. All clinical cases were primarily planned and optimised by DMPO. The set of objective values was adapted according to the clinical requirements. The head and neck cases were based on RTOG 0522 but with more demanding specifications with respect to homogeneity in the planning target volumes. Prostate patient plans were planned as described in Guckenberger et al[23]. All plans required a homogeneity expressed by a standard deviation of less than 3.5% in the central part of the PTV (which was defined as the PTV reduced by 5 mm) or the CTV. The breast case from Fogliata and Cozzi [22] was planned to fulfil the constraints cited herein as closely as possible (details see in the results section). Finally, a total of 11 up to 31 objectives for 6 – 14 considered volumes were created (see Additional file 1). Help volumes were typically defined as several concentric envelopes surrounding the PTVs, or additional structures to accentuate concave parts of the PTV, if no particular OAR was present. For all plans of a given case the identical set of objectives was used. Only objectives and no constraints were utilised for the planning procedure. This was to keep the optimisation process flexible. Mainly 4 points (2 MinDVH, 2 MaxDVH) of a DVH-curve were used to shape a PTV-DVH, 2 points to describe the shape of an OAR-curve (more, if the OAR was near to the PTV or overlapping, less, if the OAR was distant from it). The weights for the PTV were chosen between 10 and 100 to force the system to follow the objectives very tightly. The penalty of "uniform dose" as used in 10 of the cases for the (central) PTV aims at both directions, lower and higher doses. Here a weight of 1 was chosen. The objective weights of the organs at risk were mostly between 0.1 and 3, to pull the DVH-curve loosely to lower values. In summary, a dynamic range of 1000 was used for the objective weights.

If a clinical plan existed, it was used as the "reference plan". In all other cases the DMPO-25-plan served as reference (see Additional file 2: A_1_, C_1b_, C_2_, C_3b_, Q_9_, Q_15_)

## Results

Each of the optimisation runs took typically half an hour. The manual segment shape definition took between one and to two hours, depending on the complexity of the case (future computerisation should reduce this step to a couple of minutes or less).

### All entities (A-F, Q)

**Figure 2:**
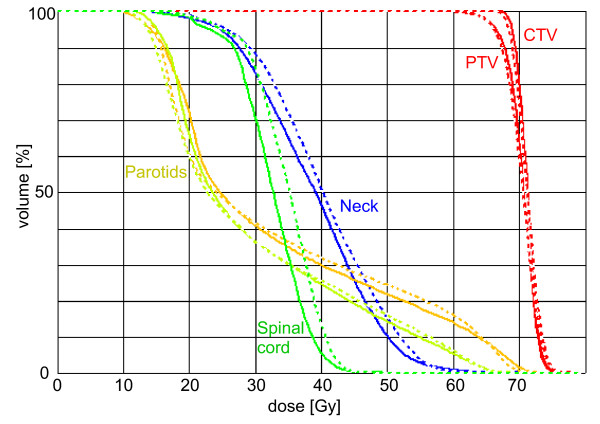
**DVH for the head and neck case B_2_**. Dose volume histogram of DMPO-50 (dashed line) and 2S-DMPO-50-technique (solid line) in the head and neck case B_2 _(see Additional file 1 and see Additional file 2) with clinical target volume (CTV), planning target volume (PTV), both parotids, spinal cord (combined with medulla oblongata) and neck.

Figure 1 depicts one typical head and neck example (B_2_) each of fluence distribution for the different planning methods, Figure 2 is added to show the referring DVH for the 2S-DMPO-50 and the DMPO-50 plan, respectively. Segment numbers, relative monitor units with respect to the reference plan and the relative "composite objective value" (COV – the sum of all weighted objectives) were shown for all cases in Tab. 2 (see Additional file 2). The relative COV was taken as a measure for the achieved quality of the plan. Based on the mean values, the 2-step IMRT pre-defined plans achieved slightly lower COV (factors 0.93 = mean (O3)/mean (O1) and 0.88 = mean (O4)/mean (O2)) for 25 and 50 steps, respectively for the same number of iteration steps. The decrease of the relative COV along the last 25 steps (from 25 to 50) was similar for DMPO and 2S-DMPO (factors 1.43 = mean (O1)/mean (O2) and 1.52 = mean (O3)/mean (O4), respectively). That means the speed of convergence is almost the same for both methods. The relative number of monitor units for 2S-DMPO was slightly higher than for DMPO (5% for 25 steps, 3% for 50 steps). In both procedural manners the monitor units tend to increase for increasing step numbers and increasing quality.

Regarding the individual cases, in 16 of 22 situations (50 steps) and 12 of 22 (25 steps) the 2S-DMPO-plan was superior to the DMPO, taken the COV as a measure of quality as to be discussed later. It can be stated that 2S-DMPO plans were equivalent with DMPO (if not better than DMPO, for 50 optimisation steps). "IMRT", the classical fluence optimisation followed by the sequencing process (data not shown in Additional file 2, but summarized here), clearly cannot compete, neither with respect to the quality (mean relative COV: 1.92), nor regarding monitor units (relative mean value: 1.33) or segment numbers (mean number of segments:143-always more segments than with any other technique used in this work). The "IMRT" results should be seen as a reference for the comparison of 2S-DMPO and DMPO: Their differences are rather small in relation to "IMRT".

A closer look at the results shows differences for the groups of cases. In all groups except B and C the 2S-DMPO-50 COV was lower than that for DMPO-50. For the breast cases (C) three 2S-DMPO-plan were not advantageous. Therefore, the breast cases were scrutinized in more detail.

### Breast case (C)

**Figure 3:**
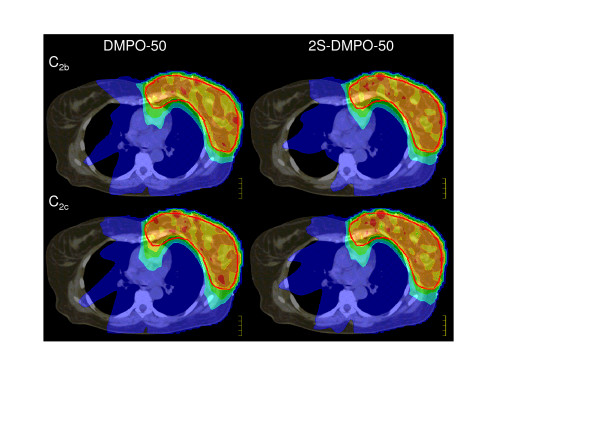
**Dose distributions for the breast case C_2_**. Dose distribution of DMPO-50 and 2S-DMPO-50-technique in a breast case C_2 _variants "b" (sparing of the contralateral lung) and "c" (sparing of the ipsilateral lung) – see Additional file 3. Patient model and objectives from Fogliata et al[22]. with kind permission of L. Cozzi. Dark blue: doses above 10%, blue: 50%, green: 70%, yellow: 90%, orange: 100%, red: 110%.

For the breast case C_2_, the parameter list from the comparison study of Fogliata et al[22] was used (the referring dose distribution of several plans to be compared with [22] is depicted in Figure 3). Following their example, the DVH of the PTV, the ipsi- and contralateral lungs, the contralateral breast, the heart and the outline without the PTV were evaluated. "Effective maximum" and "effective minimum" doses were defined there as "significant" ignoring a volume of 1.8 cm^3 ^containing the extreme doses. The focus of the plan versions a, b, c varied. "a" emphasised the homogeneity in the PTV, "c" accentuated the sparing of the ipsilateral lung, "b" was an intermediate plan version, specifically sparing the contralateral lung. In Tab. 3 (see Additional file 3) the DMPO-50 and 2S-DMPO-50 plans b, c were compared with original data from the study provided by the group of Fogliata and Cozzi. The mean values of all participant institutions and the values of the two Pinnacle3^®^-plans of the Fogliata-study are depicted for comparison. Bold numbers mark a transgression of constraints. Values worse than the mean value of the Pinnacle results from the Fogliata-study were shaded in grey. The distribution of the grey colour indicates that both, the DMPO and the 2S-DMPO plans can concur with the Pinnacle3^®^-results of the Fogliata-study. Again, no clear preference can be seen with respect to DMPO or 2S-DMPO.

Adopting the same measures to cases C_1_, C_3 _and C_4_, 2S-DMPO-50 and DMPO-50 were better in 59 and 40 instances, respectively (110 parameters in total, not shown here in detail). However, also this result can also not identify an optimal method.

### Quasimodo case (Q)

The Quasimodo [21] phantom constitutes the clearest geometrical constellation, which was challenging because the PTV partially surrounded the OAR with small distance between them. According Bohsung et al. [21] the dose is normalised to the mean dose in the PTV. The 95%-isodose should include 99% of the PTV-volume, whereas the 105%-isodose should encompass not more than 5% of the PTV-volume. The constraints concerning OAR and Body\PTV are given in Tab 4 (see Additional file 4). The quality index S_D _is defined simply as the sum of the differences of the constraints and the actual values if they violate the constraints. Ideally the S_D _is near to zero. The evaluation of the QUASIMODO-data in Tab. 4 shows notable advantage of the 2S-DMPO compared to DMPO. For 9 irradiation directions only much more segment numbers (75) of a DMPO plan lead to equivalent results as a 40 segment 2S-DMPO technique (S_D _= 6.1 vs. 6.8). For 15 gantry angles the desired quality index "0" could be achieved only by 2S-DMPO-50, indicating that the optimal conditions were almost met by 2-Step IMRT. Even 2-Step IMRT as segmentation alone with subsequent weight optimisation, without subsequent DMPO ("2S"), generated better results (S_D _= 4.6) than DMPO-25 with 25 iteration steps (S_D _= 4.9). As a secondary result, it should be noted that an increase of gantry angles – even with no increase in the number of segments (75 in both cases) – conceptually can enhance the quality of a plan.

## Discussion

### Quality of 2S-DMPO

It should be noted that on average 20 "objectives" (see Additional file 1) were used as described in the materials section to shape the DVH of each plan; all of them were weighted quadratic deviations from a desired point of a DVH curve. Based on this, the CSV is well suited to assess the quality of an approach relative to a desired DVH, encouraging the authors to utilize it to analyze DMPO and 2S-DMPO. The parameter sets were primarily developed for pure DMPO-plans; so it is debatable whether they could be advantageous for DMPO. However, besides the breast cases that needed further consideration, most other case-types benefited from a 2-Step IMRT-pre-definition of the segments.

Furthermore the comparison with the published data of other groups in the Quasimodo-project [21] confirms the high quality of the 2S-DMPO-plans – without exception all 2S-DMPO plans would be placed in the leading group of all participants, the 15-field 2S-DMPO-50 result of this study was only achieved by an intensity modulated arc technique in the Quasimodo-group. Not even a prolongation of the optimisation process to 100 steps enabled the pure DMPO plan to match 2S-DMPO-50 (result not presented here). For the 9-field plan only a drastic increase of the segment number (from 45 to 75) led to a plan of similar quality as the 9-field 2S-DMPO. This suggests that the 2-Step IMRT pre-segmentation is proximate to the theoretical optimum which could not always be met by the pure DMPO.

For the breast data 2-Step IMRT was discussed critically in former work [9,10]. Theoretical considerations [10] conjectured that for breast cases a third segment step could be favourable (that means i. e. splitting of the of 2^nd ^order segments in a broader and a narrower part). The breast results of pure 2-Step IMRT [6] were the "worst" compared to all other cases. Nevertheless 2S-DMPO was comparable to pure DMPO in this study. A detailed view on the results in Tab. 3 (see Additional file 3) shows that both methods lead to similar results; especially when considering the wide range of results delivered by different working groups with different planning systems. With respect to all values, the results from this work are qualitatively better than most from the Fogliata study; in general they are in the leading group. The mean values of this study were better for 21 out of 22 parameters than the mean values over all participant systems. The results of this study closely resembled the results of the two variants of Pinnacle-applications "Pinn1" and "Pinn2" from the Fogliata-study [22]. The "b" variants 2S-DMPO-50 DMPO-50 achieved better results in almost all categories besides the dose to the ipsilateral lung; however, the mean dose to both lungs was lower. The "c"-variants also tended to be better with respect to the lung doses and the minimum dose in the PTV with minor disadvantages with respect to hot spots in the PTV. The differences between DMPO and 2S-DMPO were much smaller within one set of optimisation parameters in contrast to the differences regarding other parameter sets or the Pinnacle3^® ^plans presented by Fogliata et al. [22] in their study. The differences within all Pinnacle3^® ^plans and the plans based on other planning systems from their study were even greater.

### Plan quality and number of gantry angles

Do more gantry angles entail more segments? It can be shown, that increasing the number of gantry angles does not increase or even reduce the necessary number of segments: In a thought experiment an IMRT plan is optimized for a given configuration of gantry angles and number segments. The optimization is performed by minimizing an objective function (OF) of arbitrary type. Now, one or more additional gantry angles should be allowed, however, with the same number of segments in total. The system gains more degrees of freedom. Nevertheless, the primary plan is one possible solution, because all the new gantry angles can be rejected without increasing the value of the OF. Perhaps another configuration leads to a lower value for the OF and therefore to a better plan. In a next step it could be tried to reduce (!) the number of segments. That is just the situation as for the Quasimodo-case: For DMPO-50 and 9 gantry angles the 75 segment plan the quality score SD = 6.1 is much higher than for 15 gantry angles and 70 segments (SD = 2.7). For this reason few gantry angles are not automatically favourable.

Also, increasing the number of beams with constant number of segments need not be more time consuming. In all cases with equidistant gantry angles, a total gantry angle of 360° (n-1)/n must be covered (n, n': number of fields). Furthermore the time loss Δt_g _per starting or stopping-process of the gantry has to be considered. Comparing two techniques changing from n to n' fields, a time loss of t = 360° (1/n' - 1/n)/v + Δt_g _(n - n') results for a gantry velocity v. On the other hand, a time gain is to be taken into account, because during the gantry motion a segment can be formed, gaining Δt_S _per such segment and overall gaining Δt_S _(n' - n) comparing the two techniques. Typical values for two types of the accelerators in our department (values of the second one in brackets) are v = 360°/78 s (360°/135 s), Δt_g _= 4 s (2 s), Δt_g _= 8.5 s (4 s), resulting in a time gain of 11 s (the other linac type: 4 s time loss). for the 9-field technique in relation to a 5-field technique with the same number of segments, monitor units and does rate. Obviously the number of gantry angles must be discussed separately. Techniques using more gantry angles are not automatically slower and could be even advantageous with respect to time efficiency.

## Conclusion

In summary, segment shape optimization with DMPO was nearly equivalent for both methods, the segment pre-definition by 2-Step IMRT and the pre-definition by inverse planning (with some advantages for 2S-DMPO). The 2-Step IMRT algorithm has been shown to be a well-suited method to pre-define segments, even for breast cases that were thought to be inappropriate in earlier studies for 2-Step IMRT alone. In all 22 cases and variants, 2-Step IMRT combined with DMPO was able to produce results which could compete with IMRT algorithms of the newest generation, i.e. DMPO alone. The quality of the plans were superior, when compared with segmentation after completion of the fluence optimisation.

One advantage of 2-Step IMRT is that it generates "intuitive" IMRT segments, reflecting the anatomy of the patient. In earlier work [9,10] the author proposed 2-Step IMRT as a segmentation method with the potential for fast day-to-dayIMRT adaptation. This feature can now be investigated in further work.

## Competing interests

The authors declare that they have no competing interests.

## Authors' contributions

KB was responsible for the primary concept and the design of the study; he performed the calculations and drafted the manuscript. JM critically reviewed the study and revised the manuscript. MF was responsible for the patients, reviewed patient data and revised the manuscript.

## Supplementary Material

Additional file 1 **Numbers of volumes and objectives.** Numbers of types of objectives and volumes of the clinical and Quasimodo cases. SIB: Simultaneous integrated boost. Click here for file

Additional file 2 **Comparison of the plan quality.** Segment numbers (columns S), Relative Monitor units (M) and relative composite objective values (O) with respect to the reference plan for clinical (A-F) and Quasimodo (Q) cases. SIB: Simultaneous integrated boost. Click here for file

Additional file 3 **Detailed quality parameters for the breast case C2.** Detailed comparison of lung-sparing variants "b" and "c" for 2S-DMPO-50 and DMPO-50. Objectives and data of study C2 from Fogliata et al. [22] (detailed information with friendly permission of L. Cozzi) Pinn 1 and Pinn 2: Pinnacle-results of the study. Bold: Objectives not met, gray-shaded: values worse than the related mean value from the Pinnacle3^®^-results in the Fogliata-study. Click here for file

Additional file 4 **Quasimodo **[21]** – objectives**. Quasimodo [21] – Objectives and the achieved values for several planning conditions. Q15: 15 equidistant gantry angles, Q9: 9 equidistant gantry angles. Body\PTV: Healthy Body, 2S: 2-Step IMRT with weight optimisation only. SD: Quality index (sum of deviations from the given constraints in the case of a violation of the related constraint). Click here for file
