# Supplementary material for: Pre-segmented 2-Step IMRT with subsequent direct machine parameter optimisation – a planning study
Source: Radiat Oncol. 2008 Nov 6;3:38. doi: 10.1186/1748-717X-3-38 (PMC2612672; doi:10.1186/1748-717X-3-38)
Supplement: Additional file 1 — Numbers of volumes and objectives. Numbers of types of objectives and volumes of the clinical and Quasimodo cases. SIB: Simultaneous integrated boost. [file 1748-717X-3-38-S1.doc]

|  |  | Number of Objectives | | | Number of Volumes | | |
| --- | --- | --- | --- | --- | --- | --- | --- |
| Type  Case | | Min-DVH | Max-DVH | Uniform | PTV | OAR | Help-volumes |
| A_1_ | SIB | 5 | 13 | 0 | 2 | 3 | 2 |
| A_2_ | SIB | 5 | 23 | 1 | 2 | 7 | 3 |
| A_3_ | SIB | 5 | 23 | 1 | 2 | 7 | 3 |
| A_4_ | SIB | 4 | 15 | 0 | 2 | 4 | 1 |
| B_1_ | SIB | 4 | 22 | 0 | 2 | 9 | 3 |
| B_2_ |  | 3 | 19 | 1 | 1 | 9 | 1 |
| B_3_ | 2 SIB | 7 | 24 | 0 | 3 | 9 | 0 |
| B_4_ | SIB | 6 | 22 | 1 | 2 | 8 | 2 |
| C_1_ |  | 1 | 10 | 0 | 1 | 4 | 1 |
| C_2_ [21] |  | 4 | 20 | 0 | 1 | 8 | 3 |
| C_3_ |  | 3 | 13 | 1 | 1 | 5 | 2 |
| C_4_ |  | 3 | 13 | 1 | 1 | 5 | 2 |
| D |  | 3 | 7 | 1 | 1 | 6 | 0 |
| E | SIB | 4 | 13 | 0 | 2 | 4 | 2 |
| F | SIB | 5 | 10 | 1 | 2 | 3 | 2 |
| Q_9_ [20] |  | 1 | 12 | 1 | 1 | 4 | 1 |
| Q_15_ [20] |  | 1 | 12 | 1 | 1 | 4 | 1 |
|  |  |  |  |  |  |  |  |
| Mean |  | 3.8 | 15.9 | 0.6 | 1.6 | 5.8 | 1.7 |
