# Supplementary material for: Pre-segmented 2-Step IMRT with subsequent direct machine parameter optimisation – a planning study
Source: Radiat Oncol. 2008 Nov 6;3:38. doi: 10.1186/1748-717X-3-38 (PMC2612672; doi:10.1186/1748-717X-3-38)
Supplement: Additional file 2 — Comparison of the plan quality. Segment numbers (columns S), Relative Monitor units (M) and relative composite objective values (O) with respect to the reference plan for clinical (A-F) and Quasimodo (Q) cases. SIB: Simultaneous integrated boost. [file 1748-717X-3-38-S2.doc]

|  |  | Segment Number | | | | Relative Monitor Units | | | | Relative Composite Objective Value | | | |
| --- | --- | --- | --- | --- | --- | --- | --- | --- | --- | --- | --- | --- | --- |
| Technique  Case | | DMPO-25 | **DMPO-50** | 2S- DMPO-25 | **2S- DMPO-50** | DMPO-25 | **DMPO-50** | 2S- DMPO-25 | **2S- DMPO-50** | DMPO-25 | **DMPO-50** | 2S- DMPO-25 | **2S- DMPO-50** |
| A_1_ | SIB | 50 | **50** | 49 | **45** | 1.00 | **1.07** | 1.48 | **1.45** | 1.00 | **0.76** | 0.72 | **0.42** |
| A_2_ | SIB | 30 | **30** | 35 | **24** | 1.05 | **1.00** | 1.56 | **1.43** | 1.17 | **1.00** | 2.11 | **1.00** |
| A_3_ | SIB | 40 | **40** | 40 | **40** | 1.13 | **1.20** | 1.25 | **1.32** | 1.14 | **0.79** | 0.96 | **0.75** |
| A_4_ | SIB | 43 | **42** | 43 | **44** | 1.03 | **1.03** | 2.03 | **2.05** | 1.60 | **0.94** | 0.96 | **0.78** |
| B_1_ | SIB | 52 | **52** | 54 | **54** | 1.03 | **1.08** | 1.08 | **1.12** | 1.00 | **0.68** | 0.71 | **0.51** |
| B_2_ |  | 52 | **52** | 53 | **51** | 0.97 | **1.00** | 1.06 | **1.03** | 1.28 | **1.02** | 1.66 | **0.83** |
| B_3_ | 2 SIB | 59 | **59** | 62 | **60** | 0.76 | **0.83** | 0.76 | **0.79** | 1.41 | **1.02** | 1.14 | **0.79** |
| B_4_ | SIB | 49 | **49** | 52 | **52** | 1.17 | **1.23** | 1.27 | **1.34** | 1.56 | **1.19** | 2.32 | **1.37** |
| C_1a_ |  | 21 | **21** | 19 | **20** | 1.77 | **2.30** | 0.93 | **0.91** | 2.03 | **0.89** | 0.82 | **0.79** |
| C_1b_ |  | 35 | **35** | 33 | **29** | 1.00 | **1.04** | 0.94 | **0.98** | 1.00 | **0.71** | 1.02 | **0.66** |
| C_2a_ [21] |  | 49 | **44** | 32 | **32** | 1.00 | **1.07** | 0.82 | **0.89** | 1.00 | **0.76** | 1.19 | **0.79** |
| C_2b_ [21] |  | 32 | **31** | 33 | **31** | 1.00 | **0.98** | 0.90 | **0.98** | 1.00 | **0.78** | 1.08 | **0.66** |
| C_2c_ [21] |  | 35 | **33** | 30 | **30** | 1.00 | **1.04** | 0.95 | **1.01** | 1.00 | **0.85** | 1.23 | **0.91** |
| C_3a_ |  | 32 | **32** | 31 | **31** | 0.71 | **0.76** | 0.86 | **0.96** | 2.70 | **1.13** | 1.90 | **0.98** |
| C_3b_ |  | 28 | **28** | 24 | **23** | 1.00 | **1.05** | 0.85 | **0.94** | 1.00 | **0.81** | 1.03 | **0.81** |
| C_4a_ |  | 50 | **50** | 46 | **41** | 1.13 | **1.18** | 0.78 | **0.87** | 1.14 | **0.94** | 1.10 | **0.83** |
| C_4b_ |  | 30 | **28** | 30 | **29** | 0.96 | **1.00** | 0.70 | **0.77** | 1.18 | **1.00** | 0.83 | **0.58** |
| D |  | 75 | **75** | 65 | **65** | 0.90 | **0.92** | 0.75 | **0.77** | 0.94 | **0.85** | 0.83 | **0.79** |
| E | SIB | 45 | **43** | 41 | **41** | 0.89 | **0.93** | 1.02 | **1.05** | 1.04 | **0.90** | 1.16 | **0.91** |
| F | SIB | 49 | **49** | 31 | **31** | 0.87 | **0.87** | 1.07 | **1.07** | 1.03 | **0.71** | 1.51 | **0.55** |
| Q_9_ [20] |  | 45 | **45** | 42 | **40** | 1.00 | **1.03** | 1.08 | **1.09** | 1.00 | **0.61** | 0.45 | **0.42** |
| Q_15_ [20] |  | 70 | **70** | 67 | **67** | 1.00 | **1.00** | 0.98 | **0.97** | 1.00 | **0.66** | 0.60 | **0.51** |
|  |  |  |  |  |  |  |  |  |  |  |  |  |  |
| Mean |  | 44 | **43** | 41 | **40** | 1.02 | **1.07** | 1.05 | **1.08** | 1.24 | **0.86** | 1.15 | **0.76** |
| Std.Dev. |  | 13 | **14** | 13 | **14** | 0.20 | **0.30** | 0.31 | **0.29** | 0.42 | **0.15** | 0.48 | **0.22** |
| Col # | | S1 | **S2** | S3 | **S4** | M1 | **M2** | M3 | **M4** | O1 | **O2** | O3 | **O4** |
