# Supplementary material for: Pre-segmented 2-Step IMRT with subsequent direct machine parameter optimisation – a planning study
Source: Radiat Oncol. 2008 Nov 6;3:38. doi: 10.1186/1748-717X-3-38 (PMC2612672; doi:10.1186/1748-717X-3-38)
Supplement: Additional file 3 — Detailed quality parameters for the breast case C2. Detailed comparison of lung-sparing variants "b" and "c" for 2S-DMPO-50 and DMPO-50. Objectives and data of study C2 from Fogliata et al. [22] (detailed information with friendly permission of L. Cozzi) Pinn 1 and Pinn 2: Pinnacle-results of the study. Bold: Objectives not met, gray-shaded: values worse than the related mean value from the Pinnacle3®-results in the Fogliata-study. [file 1748-717X-3-38-S3.doc]

|  |  | PTV V90 | PTV V95 | PTV V107 | PTV V115 | PTV Dmin eff | PTV Dmax eff | PTV Std | ipsilateral  Lung D_mean_ | ipsilateral Lung V40 | ipsilateral Lung V90 | Heart D_mean_ | Heart D_max_ eff | Heart V40 | contralateral Lung D_mean_ | Contralateral Breast D_mean_ | Contralateral Breast D_max_ eff | Contralateral Breast V20 | Healthy Tissue D_mean_ | Healthy Tissue D_max_ eff | Healthy Tissue V90 | Healthy Tissue V40 | Healthy Tissue V20 |
| --- | --- | --- | --- | --- | --- | --- | --- | --- | --- | --- | --- | --- | --- | --- | --- | --- | --- | --- | --- | --- | --- | --- | --- |
|  |  | [%] | [%] | [%] | [%] | [%] | [%] | [%] | [%] | [%] | [%] | [%] | [%] | [%] | [%] | [%] | [%] | [%] | [%] | [%] | [%] | [%] | [%] |
| *Case* | *Objective* | ***100*** | ***max*** | ***0*** | ***0*** | ***> 90%*** | ***< 107%*** | ***min*** | ***< 30%*** | ***< 22%*** | ***min*** | ***min*** | ***min*** | ***min*** | ***<  30%*** | ***< 10%*** | ***< 70%*** | ***min*** | ***min*** | ***min*** | ***min*** | ***min*** | ***min*** |
|  |  |  |  |  |  |  |  |  |  |  |  |  |  |  |  |  |  |  |  |  |  |  |  |
| Fogliata C_2_ [21] Cozzi | Overall  Mean | **94.8** | 84.2 | **10.8** | **0.9** | **67.6** | **116.2** | 6.1 | **31.6** | **26.0** | 3.3 | 29.6 | 76.1 | 20.6 | 14.5 | **10.9** | 41.7 | 10.5 | 18.5 | 116.4 | 3.8 | 17.2 | 33.5 |
|  |  |  |  |  |  |  |  |  |  |  |  |  |  |  |  |  |  |  |  |  |  |  |  |
|  | Pinn1 | **96.6** | 87.0 | **6.7** | 0.0 | **65.9** | **114.2** | 5.3 | **30.5** | 20.8 | 4.3 | 27.2 | 87.5 | 15.4 | 12.7 | 7.1 | 26.5 | 1.8 | 17.4 | 136.7 | 3.6 | 16.0 | 32.9 |
|  | Pinn2 | **96.8** | 88.8 | **6.6** | **0.3** | **67.3** | **117.0** | 5.2 | 28.7 | **22.5** | 3.7 | 25.8 | 76.0 | 11.0 | 13.4 | 8.6 | 41.1 | 6.9 | 16.2 | 121.0 | 3.1 | 14.7 | 28.9 |
|  |  |  |  |  |  |  |  |  |  |  |  |  |  |  |  |  |  |  |  |  |  |  |  |
| C_2b_ [21] | 2S-DMPO | **97.6** | 87.1 | **5.6** | **0.1** | **78.5** | **114.5** | 4.7 | **32.1** | 21.8 | 2.7 | 23.9 | 76.1 | 6.9 | 9.9 | 7.5 | 39.3 | 3.0 | 14.8 | 111.4 | 2.4 | 12.8 | 25.2 |
|  | DMPO | **97.3** | 84.7 | **6.7** | 0.0 | **78.8** | **113.8** | 5.0 | **33.0** | **23.5** | 2.8 | 21.2 | 78.3 | 6.9 | 11.2 | 7.0 | 35.8 | 4.5 | 15.6 | 111.0 | 2.2 | 13.3 | 30.2 |
| C_2c_ [21] | 2S-DMPO | **96.5** | 83.1 | **8.4** | **0.2** | **75.1** | **117.5** | 5.4 | 28.3 | 17.1 | 2.0 | 24.1 | 78.2 | 7.7 | 11.3 | 7.5 | 29.6 | 2.1 | 15.0 | 113.5 | 2.2 | 12.8 | 28.2 |
|  | DMPO | **97.1** | 85.2 | **6.2** | **0.1** | **76.3** | **115.4** | 5.0 | 28.4 | 18.2 | 2.5 | 21.5 | 74.3 | 6.4 | 10.9 | 7.2 | 26.5 | 1.8 | 15.8 | 113.0 | 2.2 | 13.6 | 30.0 |
