# Supplementary material for: Pre-segmented 2-Step IMRT with subsequent direct machine parameter optimisation – a planning study
Source: Radiat Oncol. 2008 Nov 6;3:38. doi: 10.1186/1748-717X-3-38 (PMC2612672; doi:10.1186/1748-717X-3-38)
Supplement: Additional file 4 — Quasimodo [21] – objectives. Quasimodo [21] – Objectives and the achieved values for several planning conditions. Q15: 15 equidistant gantry angles, Q9: 9 equidistant gantry angles. Body\PTV: Healthy Body, 2S: 2-Step IMRT with weight optimisation only. SD: Quality index (sum of deviations from the given constraints in the case of a violation of the related constraint). [file 1748-717X-3-38-S4.doc]

|  |  |  |  | **Constraints [%]** | | | | | |  |
| --- | --- | --- | --- | --- | --- | --- | --- | --- | --- | --- |
|  | Technique | Segment number | Monitor units | PTV  V_95_ | PTV  V_105_ | OAR  V_70_ | Body\PTV V_80_ | Body\PTV V_100_ | Body\PTV D_max_ | S_D_ |
|  |  |  |  | **>99** | **<5** | **<1** | **<15** | **<2** | **<105** | **0** |
| Q_15_ | “IMRT” | 172 | 681 | 97.7 | 0.0 | 0.1 | 12.7 | 0.6 | 101.8 | 1.3 |
|  | DMPO-25 | 75 | 633 | 97.7 | 2.6 | 0.3 | 11.2 | 0.9 | 108.6 | 4.9 |
|  | **DMPO-50** | **70** | **636** | **98.3** | **0.4** | **0.2** | **10.1** | **0.4** | **106.0** | **2.7** |
|  | 2S | 75 | 625 | 96.4 | 3.5 | 0.0 | 11.4 | 0.3 | 107.0 | 4.6 |
|  | 2S-DMPO-25 | 67 | 619 | 99.3 | 0.5 | 0.2 | 11.6 | 0.3 | 105.8 | 0.8 |
|  | **2S-DMPO-50** | **67** | **612** | **99.1** | **0.0** | **0.2** | **11.4** | **0.2** | **104.2** | **0** |
| Q_9_ | “IMRT” | 99 | 628 | 96.8 | 0.9 | 0.1 | 12.1 | 1.1 | 110.0 | 8.2 |
|  | DMPO-25 | 45 | 547 | 94.1 | 7.0 | 1.9 | 11.7 | 0.9 | 109.9 | 12.7 |
|  | **DMPO-50** | **45** | **564** | **94.6** | **5.4** | **0.6** | **11.0** | **0.6** | **110.6** | **10.4** |
|  | DMPO-25 | 75 | 577 | 95.3 | 4.5 | 1.2 | 11.4 | 1.0 | 110.8 | 9.7 |
|  | DMPO-50 | 75 | 610 | 96.7 | 4.3 | 0.5 | 10.2 | 0.5 | 108.8 | 6.1 |
|  | 2S | 45 | 588 | 93.7 | 7.8 | 0.9 | 12.7 | 0.7 | 112.4 | 15.5 |
|  | 2S-DMPO-25 | 42 | 589 | 97.6 | 5.8 | 0.5 | 12.8 | 0.8 | 109.7 | 6.9 |
|  | **2S-DMPO-50** | **40** | **596** | **97.7** | **5.4** | **0.2** | **12.8** | **0.8** | **110.1** | **6.8** |
